# Bilirubin as a Diagnostic Factor in Acute Appendicitis

**DOI:** 10.7759/cureus.93777

**Published:** 2025-10-03

**Authors:** Abdelrahman Elafandy, Sandeep Singh

**Affiliations:** 1 General Surgery, Warwick Hospital, Warwick, GBR

**Keywords:** acute abdomen, appendicitis, bilirubin, hyperbilirubinemia, right iliac fossa pain

## Abstract

Background: Acute appendicitis is one of the most common surgical emergencies. The decision for surgical management can be challenging, especially in young patients and in females. There have been many trials to have a scoring system to help the surgical decision with many laboratory and clinical inputs, such as WBC count, C-reactive protein (CRP), and fever.

Objective: In this study, we tried to determine if bilirubin could be used as a diagnostic factor in acute appendicitis to help make the surgical decision.

Methods: A retrospective observational study was conducted, including all the patients who presented to Warwick Hospital’s general surgery on-call service between January and May 2024 with a complaint of acute right lower abdominal pain. All the data from patients’ records underwent data analysis.

Results: Around 360 patients were identified (n=360), 103 (28.6%) had an appendectomy, 36 (10%) of all the patients had hyperbilirubinemia, and 26 (72.2%) of the patients who had an appendectomy had hyperbilirubinemia. Bilirubin has a specificity of 96.2% and a sensitivity of 27.7% for the diagnosis of acute appendicitis in the acute presentation.

Conclusions: Bilirubin can be used as a highly specific factor for the diagnosis of acute appendicitis in acute right lower abdominal pain to support the surgical decision and to prevent the complications caused by the delay in the surgical management.

## Introduction

Acute appendicitis remains one of the most common surgical emergencies worldwide, with approximately 40,000 hospital admissions annually in the United Kingdom alone [1]. Diagnosis traditionally relies on a combination of clinical examination and basic blood tests, including total leukocyte count (TLC) and C-reactive protein (CRP) [1-3]. 

Although many patients present with classical features, such as migrating pain from the periumbilical region to the right iliac fossa, atypical presentations are common and can pose a significant diagnostic challenge, especially in the elderly and children [4]. An example is general abdominal pain in children or lower abdominal pain with diarrhea in pelvic appendicitis. Conditions such as acute ovarian pathology, diverticulitis, endometriosis, and Crohn’s disease may mimic appendicitis, further complicating the clinical picture [4]. 

Computed tomography (CT) is considered the gold standard for diagnosis, offering a sensitivity of 94% and specificity of 95% [1]. However, its use is limited in young patients and during pregnancy due to radiation exposure. Ultrasonography serves as a safer alternative but is highly operator-dependent, with reported sensitivity and specificity of 86% and 81%, respectively [1,2,5]. 

To improve diagnostic accuracy and reduce unnecessary surgeries, researchers continue to seek reliable biomarkers. While TLC and CRP are routinely used, recent evidence suggests that serum bilirubin, particularly in complicated appendicitis, may offer additional diagnostic specificity [2]. 

The pathophysiological mechanism underlying hyperbilirubinemia in appendicitis is thought to involve bacterial translocation and systemic inflammation. Some studies propose that microorganisms such as *E. coli *disrupt liver sinusoids, impairing bilirubin metabolism [3]. Others attribute elevated bilirubin to the effects of inflammatory mediators, such as nitric oxide and interleukins, on hepatobiliary function [3,5]. 

Delayed intervention in acute appendicitis increases the risk of serious complications, including perforation and gangrenous transformation [4]. MoulaBux et al. reported hyperbilirubinemia in 100% of patients with gangrenous appendicitis, highlighting its potential as a predictive marker for disease severity [2]. Similarly, Kar et al. found that hyperbilirubinemia had a positive predictive value of 97.01% for appendiceal perforation, albeit with variable sensitivity and specificity [3]. 

In this study, we aimed to evaluate the diagnostic utility of serum bilirubin in patients presenting with acute right lower abdominal pain suspected of being appendicitis. We hypothesize that this readily available laboratory parameter may enhance diagnostic confidence, facilitate timely surgical decision-making, and help reduce complications associated with delayed treatment, particularly in resource-limited settings. 

## Materials and methods

Study design and setting 

A retrospective observational cohort study was conducted at Warwick Hospital, a secondary care center within the South Warwickshire NHS Trust. The study included all consecutive patients who presented to the general surgery on-call service with a primary complaint of acute right lower abdominal pain, suspected to be appendicitis, between January 1, 2024, and May 31, 2024. Patients were identified from the emergency department, the surgical assessment unit, the pediatric assessment unit, and inpatient referrals. 

Participants and sample size 

The initial screening identified 360 patients (n=360) who met the broad inclusion criteria of acute abdominal pain. This sample size was determined by the total number of eligible presentations during the five-month study period. 

Data collection and sources 

Data were meticulously extracted from a combination of electronic and paper-based hospital records to ensure comprehensive data capture. The primary sources were the daily on-call handover lists, the hospital's electronic patient record (EPR) system, the theater management system (ORMIS), and the hospital's information database for histopathology results. For each patient, we noted the age, gender, and the site and nature of pain. 

Laboratory values included the initial WBC, neutrophil count, CRP level, and total serum bilirubin level (measured in mmol/L). Hyperbilirubinemia was defined as a bilirubin level ≥21 mmol/L. Radiological findings included results of any ultrasonography or CT scans performed. Surgical and outcome data included whether an appendectomy was performed, the time to surgery, the intraoperative findings, the final histopathology report, and the total length of hospital admission. 

Patients of all ages were included if they presented with an acute history (≤7 days) of pain localized to the right iliac fossa, the right lower abdomen, or, in the case of children, central or lower abdominal pain that led to a surgical referral to rule out appendicitis. 

We excluded patients with chronic abdominal pain (>7 days), those who underwent an elective appendectomy for an incidental finding or a suspected neoplasm, and those whose primary complaint was pain in the right upper quadrant, left iliac fossa, or the back. 

Statistical analysis 

Data were compiled and analyzed using Microsoft Excel 2007 (Microsoft Corporation, Redmond, Washington, United States). Categorical variables, presented as frequencies and percentages, were analyzed using the Chi-square test to determine the association between hyperbilirubinemia and appendicitis. The diagnostic performance of hyperbilirubinemia was evaluated by calculating its sensitivity, specificity, positive predictive value (PPV), negative predictive value (NPV), positive likelihood ratio (LR+), and negative likelihood ratio (LR-) with 95% confidence intervals (CI). A p-value of less than 0.05 was considered statistically significant for all two-tailed tests. 

## Results

Around 360 patients were identified (n=360). The demographics are demonstrated in Table 1. 

**Table 1:** Patient demographics.

Characteristic	Value
Mean age +/- SD	35.03 years +/- 15.97
Gender	
Males	161 (44.7%)
Females	199 (55.3%)
Average period of hospital admission +/- SD	
All patients	3.33 days +/- 2.97
Hyperbilirubinemia patients	5 days +/- 3.6

**Figure 1:**
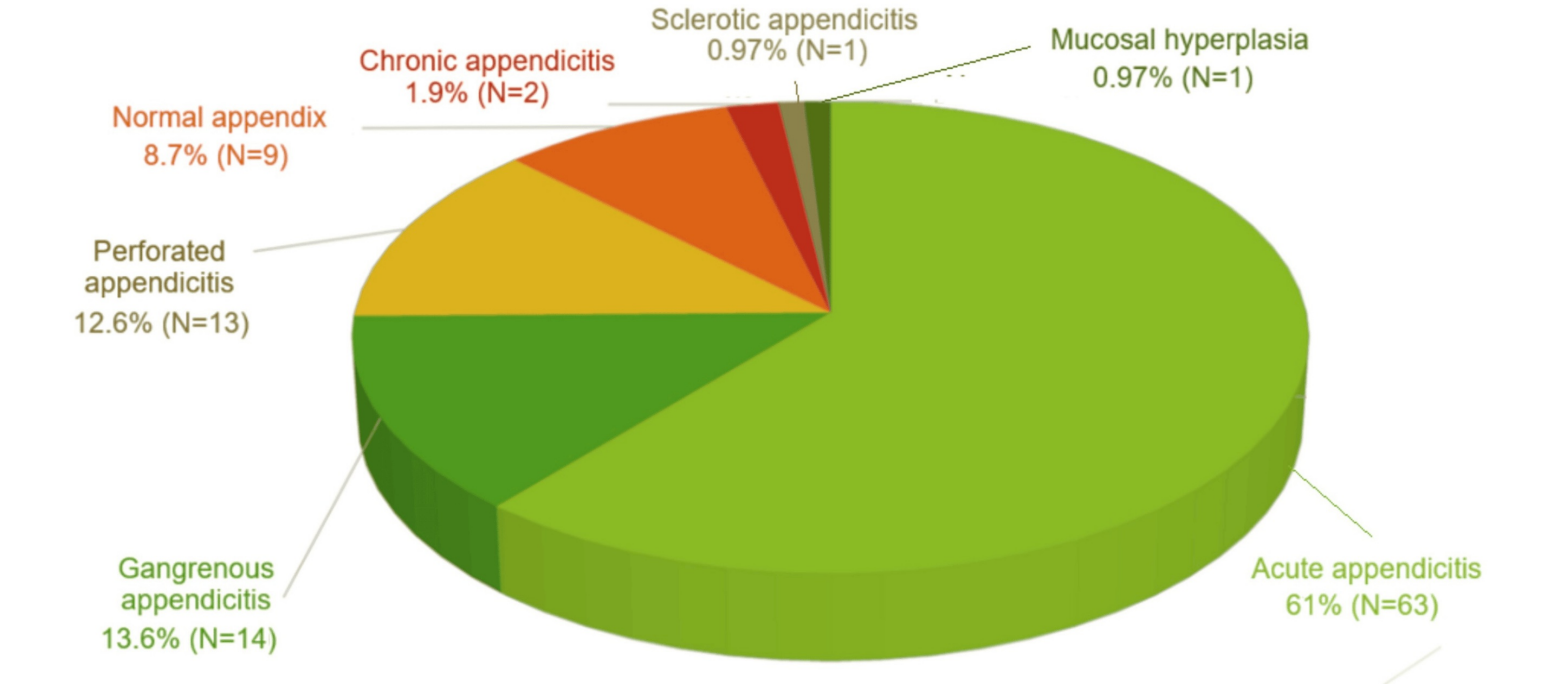
The histopathology results for patients who had appendectomy (N=103).

While 71.4% of the patients (N=257) were not treated for appendicitis or had another diagnosis, 28.6% of the patients (N=103) had an appendectomy for suspected or scan-confirmed appendicitis. Of those who had a laparoscopic appendectomy, 91.3% (N=94) were found positive for appendicitis or complicated appendicitis in the histopathology analysis; nevertheless, 8.7% (N=9) were found negative for appendicitis (Figure 1). 

The complicated appendicitis (perforations and gangrenous) was identified in about 26% (N=27) of all the patients who had appendectomy. 

**Figure 2:**
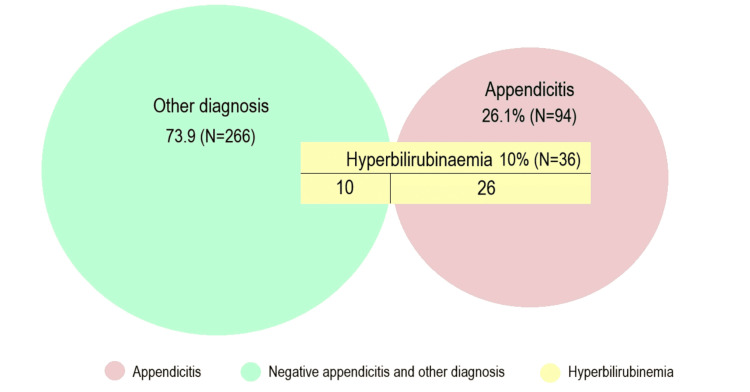
The interpretation of hyperbilirubinemia identified in all patients (n=360).

Hyperbilirubinemia was detected in 10% (N=36) of all the patients (n=360) and in 27.7% (N=26) of the patients who had positive appendicitis in the histopathology (N=94), Figure 2. 

The other 10 patients with hyperbilirubinemia (but not appendicitis) were diagnosed as demonstrated in Table 2.

**Table 2:** Hyperbilirubinemia with a diagnosis other than acute appendicitis (N=10).

Patients’ number	Diagnosis	Bilirubin level
3	Chronic hyperbilirubinemia	31,28,26
1	Crohn’s	33
1	Endometriosis	22
1	Pneumonia	28
1	Cholecystitis	31
1	Polycystic ovary disease	25
1	Diverticulitis	31
1	Superior mesenteric artery thrombosis	177

All 10 patients were diagnosed without surgery as they were either by clinical examination, unlikely to have appendicitis, or by having a scan that showed a different diagnosis. 

**Figure 3:**
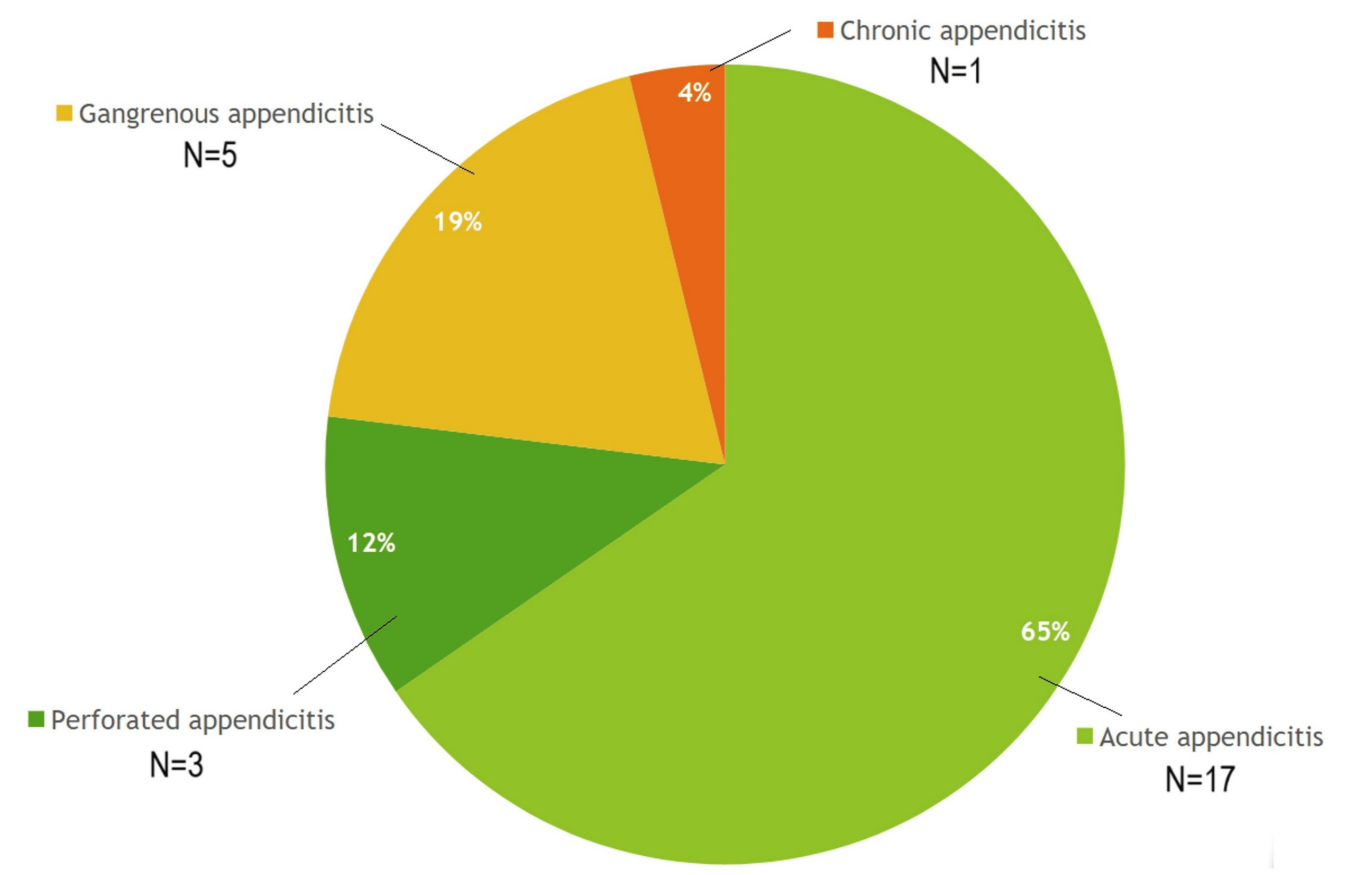
The histopathology results for patients with hyperbilirubinemia, who had appendectomies (N=26).

Around 100% of the patients with hyperbilirubinemia who underwent appendectomy had a positive histopathology for appendicitis (Figure 3). Around 19% of them had gangrenous appendicitis, and 12% had perforated appendicitis. While the majority had uncomplicated appendicitis (65%). 

As demonstrated in Table 3, the data were analyzed to calculate the sensitivity and specificity. In the Table, appendicitis points to the cases that were found positive for the disease in the histopathological analysis, either simple or complicated. While no appendicitis points to the cases with negative histopathology or those who had other diagnoses.

**Table 3:** The relation between appendicitis and the bilirubin levels. TP: true positive; FP: false positive; FN: false negative; TN: true negative

Level	Appendicitis	No appendicitis	Total
Hyperbilirubinemia >/= 21 mmol/L	26 (TP)	10 (FP)	36
Normal bilirubin	68 (FN)	256 (TN)	324
Total	94	266	360

$Sensitivity=\frac{TP}{TP+FN}=\frac{26}{26+68}=\frac{26}{94}\simeq 27.7\%$

$Specificity=\frac{TN}{TN+FP}=\frac{256}{256+10}=\frac{256}{266}\simeq 96.2\%$

As calculated from Table 3, serum bilirubin has a sensitivity of 27.7% and a specificity of 96.2% in the diagnosis of acute appendicitis. The calculated P value is < 0.00001 (Table 4). 

**Table 4:** The statistical parameters. All statistical tests were two-tailed with significance at p<0.05. Confidence intervals (CI) were calculated at 95%.

Diagnostic measure	Value (95% confidence interval)
Sensitivity	27.7% (19.2% - 37.8%)
Specificity	96.2% (93.1% - 98.1%)
Positive predictive value (PPV)	72.2%
Negative predictive value (NPV)	79.0%
Positive likelihood ratio (LR+)	7.29
Negative likelihood ratio (LR-)	0.75
Odds ratio	9.79 (4.42 - 21.68)
Chi-square (χ²) value	44.08
p-value	< 0.00001

Another interesting finding is that the average hospital stay for patients with hyperbilirubinemia was five days, while it was 3.3 days for patients with a normal bilirubin level (Table 1). We did not analyze the values of CRP and TLC as they are already established routine parameters used in daily practice. 

## Discussion

The timely and accurate diagnosis of acute appendicitis remains a critical objective in emergency surgery, directly influencing patient outcomes by mitigating the risk of severe complications such as perforation and sepsis [1]. While clinical examination and established biomarkers like leukocytosis and CRP form the diagnostic cornerstone, their limitations in atypical presentations necessitate the exploration of supplementary indicators [1,6]. This study contributes to the evolving diagnostic landscape by evaluating the role of hyperbilirubinemia, a marker increasingly associated with appendiceal inflammation, in a cohort of patients presenting with acute right lower abdominal pain. 

The association between acute appendicitis and elevated serum bilirubin is underpinned by a compelling pathophysiological mechanism. The inflammatory cascade and bacterial translocation from an inflamed appendix are believed to instigate a portal bacteremia [2]. This process, often involving organisms such as *Escherichia coli*, leads to endotoxin-mediated dysfunction of hepatobiliary transporters, ultimately impairing bilirubin excretion and resulting in its systemic elevation [2,4]. This model posits hyperbilirubinemia not as a random occurrence but as a direct physiological consequence of advanced appendiceal inflammation. 

Our findings, which demonstrate a high specificity (96.2%) for hyperbilirubinemia, strongly support its role as a marker of disease severity rather than a sensitive early indicator. This aligns with the work of MoulaBux et al. and Bakshi et al., who reported significantly elevated bilirubin levels in patients with gangrenous or perforated appendicitis compared to those with simple inflammation [2,4]. The high specificity underscores its utility in "ruling in" disease, particularly complicated cases, as a markedly elevated bilirubin level is uncommon in other benign causes of abdominal pain, thereby increasing diagnostic certainty when present. 

The diagnostic value of hyperbilirubinemia can be further quantified. Kar et al. identified a bilirubin level >1.3 mg/dL (22.2 µmol/L) as a significant threshold, carrying a PPV of 84% for appendiceal perforation [3]. This quantifiable risk stratification is crucial for surgical decision-making.

It is imperative to emphasize that hyperbilirubinemia should not be interpreted in isolation. Its limited sensitivity renders it unsuitable as a rule-out test. Instead, its primary strength lies in its integration into a multimodal diagnostic strategy. Traditional markers like leukocytosis and CRP maintain higher sensitivity for initial detection, while the addition of bilirubin enhances specificity, particularly for identifying complicated cases [1,6]. This synergistic approach allows clinicians to triangulate a diagnosis with greater confidence, especially in equivocal scenarios. 

The high specificity of hyperbilirubinemia makes it particularly valuable for risk stratification in cases where clinical and radiological findings are inconclusive. Khalid et al. advocated that elevated bilirubin levels should heighten clinical suspicion for complicated appendicitis, potentially prompting expedited surgical consultation and intervention [7]. In such contexts, this readily available laboratory value can serve as a decisive factor, reducing delays to surgery for high-risk patients and potentially improving their postoperative outcomes. 

The future of appendicitis diagnosis may lie in the development of sophisticated biomarker panels. Shuaib et al. demonstrated that combining hyperbilirubinemia with hyponatremia improved the predictive accuracy for perforated appendicitis beyond that of either marker alone [5]. This suggests that a multi-parametric model, incorporating several biomarkers each contributing a piece of the diagnostic puzzle, could achieve a level of accuracy unattainable by any single test. Such models represent a significant advancement over reliance on isolated laboratory values. 

Beyond its diagnostic role, hyperbilirubinemia may serve as an effective tool for preoperative risk stratification and surgical planning. The significant association we observed between elevated bilirubin and a longer hospital stay underscores its prognostic value, suggesting a more severe disease course and complex postoperative recovery. This is consistent with the findings of Emmanuel et al. [8], who reported that hyperbilirubinemia was a strong independent predictor of complicated appendicitis and was associated with higher rates of postoperative complications. The ability to preoperatively identify such high-risk patients allows for better resource allocation, such as ensuring senior surgical oversight, scheduling surgery on a timely basis, and preparing for potential intraoperative challenges, ultimately aiming to improve patient safety and outcomes. 

The consistent demonstration of high specificity across studies, including ours, strengthens the argument for standardizing the use of bilirubin in clinical scoring systems. The Appendicitis Inflammatory Response (AIR) score and other similar algorithms primarily rely on clinical symptoms, leukocytosis, and CRP. Integrating hyperbilirubinemia could enhance their predictive power for complications. A meta-analysis by Giordano et al. [9] concluded that hyperbilirubinemia was a significant predictor of perforation and gangrene, advocating for its inclusion in future diagnostic protocols. Developing a validated, multimodal score that incorporates this easily measurable biomarker could lead to more standardized, objective, and accurate diagnostic pathways across different clinical settings. 

Looking forward, the integration of biomarkers like bilirubin into artificial intelligence (AI) and machine learning algorithms presents a transformative frontier. Issaiy et al. conducted a systematic review highlighting that AI models incorporating blood biomarkers, including bilirubin, showed superior diagnostic and prognostic performance for acute appendicitis, with some models for predicting perforation achieving area under the curve (AUC) values exceeding 0.90 [10]. These tools can analyze complex, non-linear relationships between variables, potentially standardizing diagnostics and reducing reliance on operator-dependent imaging. 

While serum biomarkers like bilirubin enhance diagnostic confidence, their interpretation must always occur within the broader clinical context, where radiological imaging provides indispensable complementary information. Borruel Nacenta et al. [11] emphasize that modern cross-sectional imaging, particularly CT, remains the benchmark for confirming appendicitis, assessing severity, and, crucially, identifying alternative pathologies that can mimic its presentation. Their review highlights that the diagnostic value of imaging extends beyond simply visualizing an inflamed appendix; it is critical for detecting complications like perforation or abscess formation and for excluding other conditions such as diverticulitis, terminal ileitis, or gynecological disorders. Therefore, hyperbilirubinemia should be viewed as a potent biochemical red flag that can guide the judicious use of imaging, helping to prioritize patients for whom further radiological investigation is most warranted, rather than as a replacement for it. 

Furthermore, the significant correlation we observed between hyperbilirubinemia and a prolonged hospital admission period resonates with broader clinical findings. Kwon et al. demonstrated that hyperbilirubinemia in hospitalized patients is independently associated with increased mortality and morbidity, suggesting it is a marker of systemic illness severity that predicts a more complex recovery [12]. 

A principal limitation of utilizing hyperbilirubinemia is its lack of specificity for appendicitis. Its diagnostic reliability can be confounded by pre-existing conditions such as hepatic dysfunction or hemolytic disorders [1,12]. Furthermore, as its elevation is predominantly linked to advanced inflammation, its utility in detecting early, simple appendicitis is limited. Future research must focus on establishing population-specific diagnostic thresholds and investigating whether direct (conjugated) bilirubin offers superior diagnostic performance by providing a more direct measure of hepatobiliary dysfunction [3,7]. 

In conclusion, this study reinforces the growing body of evidence that hyperbilirubinemia serves as a highly specific biomarker for acute appendicitis, particularly in its advanced stages. While its low sensitivity precludes its use for screening, its value is paramount as an adjunctive tool within a multimodal diagnostic framework. It enhances risk stratification, aids in identifying complicated cases, and provides prognostic information. Future efforts should be directed toward validating combined biomarker models and integrating them into AI-driven clinical decision support systems to optimize diagnostic pathways and improve patient care. 

## Conclusions

Hyperbilirubinemia serves as a highly specific, but poorly sensitive, diagnostic marker for acute appendicitis in patients presenting with acute right lower abdominal pain. While it cannot replace comprehensive clinical assessment or diagnostic imaging, it can function as a valuable adjunct that can enhance diagnostic confidence when interpreted alongside other biomarkers and clinical findings. 

To definitively establish its role in clinical decision-making, further research is warranted. Multi-centric prospective studies are needed to validate these findings in variable populations and to determine an evidence-based, optimal cutoff value for serum bilirubin that may be tailored to different patient demographics. 
